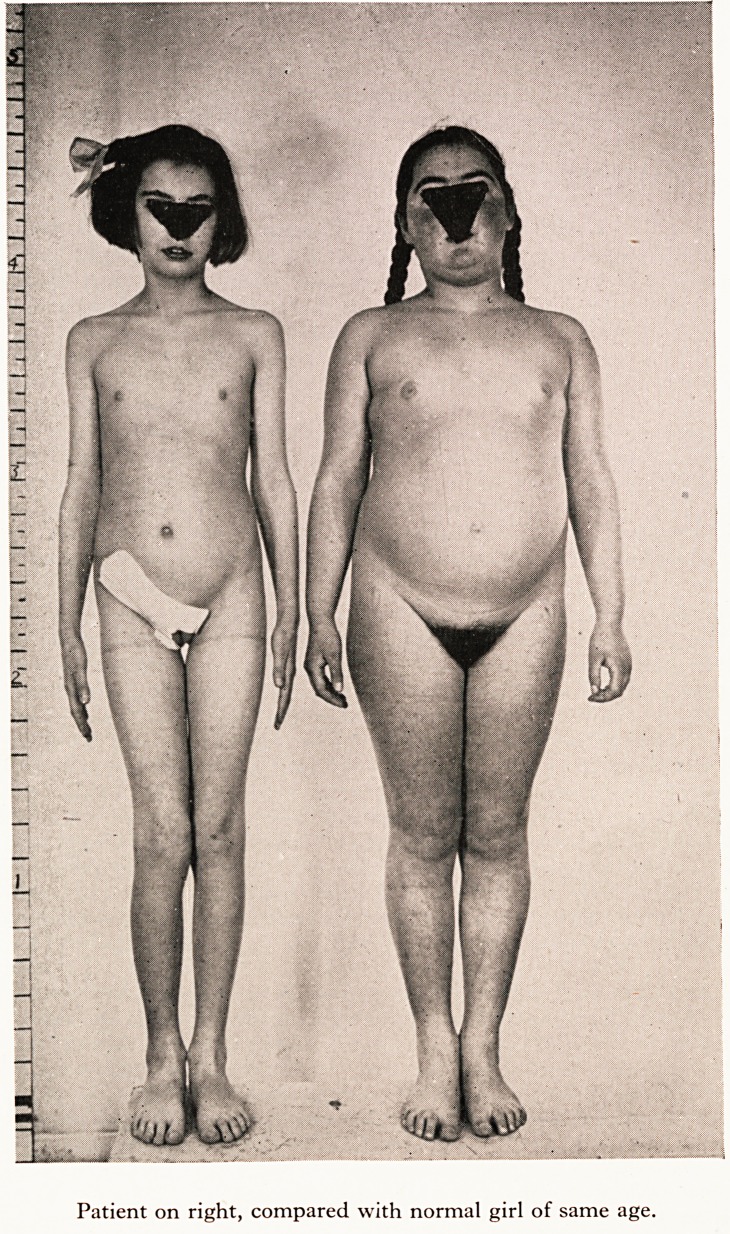# Unexplained Pyrexia in a Child

**Published:** 1952-04

**Authors:** 


### UNEXPLAINED PYREXIA IN A CHILD

FROM

### CARCINOMA OF THE ADRENAL CORTEX

*Case Report at a Clinico-Pathological Conference, Department of Pathology, University of Bristol*

**Dr. Mary Boyd** gave the clinical details:—This girl, aged eleven years and three months, was admitted to the Bristol Children's Hospital under Professor A. V. Neale in November 1951.

In June 1945, at the age of four years and nine months, she developed fever, 100 to 101 degrees in the morning rising to 104 degrees at night. This lasted three days and recurred at short intervals for three months. She also complained of abdominal pain. In February 1946, at the age of five years and five months, she had similar attacks of fever with pain across the abdomen and down the left side. The abdomen was noted to be rather larger than usual. In the winter of 1947 she again had fever and left upper abdominal pain. In November 1950, at the age of ten years and two months, pain recurred in the abdomen, which was very distended. She weighed fifty-five lb. and began to put on weight rapidly. Her waist increased two inches in one week. In April 1951 she was taken to the doctor as her relatives were questioning whether she had a glandular disorder.

In the six months before admission she developed hirsuties of upper lip, eyebrows, back and pubic region. Acne appeared over the face and upper arms and later there was backache. In the last year her weight increased from fifty-five lb. to 112 lb. and her waist measurement increased ten inches. She did not menstruate. Her general health remained good all through and her appetite was good, but recently she had a sense of fullness after small meals.

She was an obese girl (see Plate I) with plethoric complexion and acne of forehead, nose, chin and both upper arms, mild hirsuties of upper lip, eyebrows almost joined, a little axillary hair, well-developed pubic hair in female pattern and excess of downy hair on back and limbs. Height 51½ in. (normal average 57 in.); weight 112 lb. (normal average 79 lb.) There were no striae and little breast tissue, but excess of fat around the breasts and clavicular regions, as well as over the trunk in general. The waist measurement was 34 inches. There was some enlargement of the clitoris.

A mass was palpable in the left hypochondrium about three finger-breadths below the costal margin. It was tender, dull on percussion and moved a little on respiration, but its edge felt thicker and more rounded than the spleen. The apex beat could not be palpated on account of the thick chest wall. The heart sounds were normal, and the blood pressure 136/70.

The laboratory investigations were as follows: R.B.C. 5·3 million; Hb. 106 per cent; serum electrolytes all normal; serum protein 7·9 per cent. Urinary 17-ketosteroids 206 mgm. in a day (normal at this age under 5 mgm. daily), with urine volume 1,450 mls. that day.

**Dr. Jean Sheach** demonstrated X-rays:—The skull showed a normal sella turcica. In the left hypochondrium there was a soft tissue opacity with irregular calcification. An I.V.P. showed downwards and forwards displacement of the left kidney with calyces and pelvis normal. The diaphragm was elevated on the left and the heart slightly enlarged. There was no change in the lungs. The lumbar spine and femora showed no metastases.

**Mr. R. E. Horton** outlined the findings at the operation on December 13th, 1951:—The incision was made through the tenth left intercostal space, with division of the posterior ends of the tenth and eleventh ribs, and extended well forward to the mid-line of the

PLATE I

Patient on right, compared with normal girl of same age.

epigastrium. The diaphragm was divided and the pleura opened. The tumour displayed was dark red with large veins coursing over the surface. It was soft and diffluent and had undergone recent haemorrhage into its substance, which added to the difficulty of handling. The tumour was mobilized with the left kidney to which it was attached. A tongue of growth was felt extending to and probably across the mid-line. It was realized at this stage that the tumour could not be removed completely. Further, the child was now in a state of profound adrenal shock as a result of impaired function or atrophy of the other adrenal. That part of the tumour which had been mobilized was removed with the kidney. A large piece of tumour was left behind.

The girl was very cyanosed at the end of the operation, during which she had two pints of blood, methedrine and eucortone. She was given an intravenous drip of nor-adrenaline and also eucortone (10 cc. four hourly), and was placed in an oxygen tent. The blood-pressure was raised from about 50/30 to 90/64 but her general condition gradually deteriorated until she died on December 16th, 1951.

**Dr. O. C. Lloyd's** report on the operation specimen was as follows:—The specimen received consisted of the anterior half of the left kidney and of the tumour (14 by 10 by 4 cm.) at its upper pole. This tumour was soft, cream-coloured, necrotic and haemorrhagic. It was invading the renal capsule but not the kidney, and was apparently replacing the left adrenal. It was lobulated, the lobules being surrounded by fibrous capsules, which sections show are being invaded by tumour cells.

A separate half nodule (3·5 by 2·5 by 1 cm.) resembled a lymph-node metastasis, but sections showed no lymphoid tissue.

The tumour was composed of medium-sized polygonal cells, even in size but not in shape, with large round vesicular nuclei and dense basophilic cytoplasm in which there were a few small vacuoles. The cells had distinct outlines and were distributed around the blood vessels; the other areas being necrotic. Mitoses were numerous.

I think there can be little doubt that this is a cortical carcinoma of the adrenal, both on clinical grounds and because the histopathological alternatives of neuroblastoma and Wilms' tumour can be ruled out.

**Dr. G. A. C. Summers** reported the autopsy:—The external features have already been described. In addition to great obesity there was marked oedema of the back, but not of the legs or ankles. Internally there was extensive peritoneal ecchymosis, a little free blood in the peritoneal cavity and some collapse of the lower lobe of the left lung with a few recent adhesions. I soon found why total extirpation of the tumour had been impossible. It is true that the left kidney and a mass of left adrenal had been removed, but there was still a great deal of tumour tissue left. This had roughly the shape of a large thyroid gland. The left " lobe " measured 8 by 5 by 3 cm. and its lateral surface, where the surgeon's knife had passed, was rough and vascular; but only very little blood had escaped since the operation. The middle and right " lobes " were in the left renal vein and in the inferior vena cava, distending both, the former to a diameter of 3 cm., and the latter to measure 4·5 cm. from side to side and 3 cm. from front to back where the left renal vein entered. The right lobe extended 1 cm. below the lower margin of the left renal vein, and for 10 cm. from there right up behind the liver. The histological picture of the tumour has already been described.

There was considerable distension of the inferior hemi-azygos vein and of the azygos vein, which compensated for obstruction of the inferior vena cava. The heart weighed 250 g., which is twice the normal weight at this age. This was due to left ventricle hypertrophy. The lungs were oedematous and contained many small scattered metastatic deposits, with one large mass of tumour blocking and distending the main left pulmonary artery. There were no metastases in lymph-nodes nor in the liver. Microscopically there was widespread centri-lobular necrosis of the liver.

The ovaries measured 2·5 by 1·7 by 1·4 cm., and contained many follicular cysts, from 0·2 to 0·4 cm. in diameter. The Fallopian tubes and uterus were underdeveloped for the age and the endometrium was smooth with poor glandular development. The thyroid was well developed but, microscopically, activity appeared subnormal. The pituitary was of average size and shape with no histological abnormality, the basophils in particular showing no change. Inside the skull there was a narrow protuberance arising from the ethmoid bone behind the crista galli, but the frontal and other bones were normal.

The remaining adrenal was very small, only 1·5 by 1 by 0·15 cm. Microscopically the cortex was poorly developed, the zona reticularis being absent.

Several factors contributed to her death. There was a massive tumour embolism of the left pulmonary artery. Hypotension followed removal of the large actively hormone-producing tumour. The degree of centrilobular necrosis in the liver suggests that hypotension persisted after the operation in spite of therapy. The right adrenal was atrophied and incapable of taking over the function of the partly extirpated tumorous gland.

It seems that the effects of hyperadrenalism were not mediated by the pituitary as there was no abnormality of its basophil cells. Thyroid activity was apparently depressed. Certainly the ovaries were influenced, probably by an excess of follicle-stimulating hormone, which could have come from the tumour. The oestrogenizing influence of the ovaries was neutralized by the androgens of the adrenal tumour. Evidence of virilism was present in the enlarged clitoris, the florid skin, acne and abnormal 17-ketosteroid excretion. Obesity was obvious. Another point of interest was the left ventricular hypertrophy suggestive of hypertension.

**Professor T. F. Hewer:**—I think one of the most interesting features is the atrophy of the contralateral adrenal which usually occurs when there is great pathological overactivity of one gland. The removal of so much of the tumour induced what amounted to an Addisonian crisis.

**Dr. G. L. Foss:**—The technical difficulties involved in surgery of the adrenal are well recognized and the risk of death from adrenal cortical insufficiency is very great owing to the frequent atrophy of the contralateral gland. The importance of adequate supportive therapy is perhaps not appreciated as widely as it might be, but this has recently been stressed by Waltman Walters in his Moynihan lecture (1952). The most important factor is the anticipation, prevention and control of post-operative adrenal cortical insufficiency, and he recommends 200 mgm. cortisone daily for two days before and several days after the operation. Before the availability of cortisone, satisfactory results were obtained from D.O.C.A., cortical extract and glucose saline but the important point is that such therapy should be initiated *before* operation. The relative importance of collapse from removal of the sole source of nor-adrenaline is probably less.

No hard and fast rule can be laid down in correlating the clinical, biochemical and pathological findings in tumours of the adrenal cortex and in part these depend on the sex and age of the patient.

The adrenal cortex is a very complex factory, producing numerous steroids, twenty-seven of which have been isolated. These are usually grouped into those controlling electrolytes, those controlling carbohydrate and protein metabolism, and the sex hormones; these sex hormones include androgens, oestrogens and progesterone.

The clinical picture then resulting from a tumour or hyperplasia will depend on the predominance of one or more of these groups and every gradation from Cushing's syndrome to the adrenogenital syndrome may be seen.

In some prepubertal girls adrenocortical tumours producing excessive oestrogens give rise to precocious puberty and menstruation, and rarely in adult males feminism is seen from the same cause.

In this particular case the high value of 17-ketosteroids, or androgen excretion products, is in keeping with the stunted growth, acne, and other evidence of virilism, but her obesity and plethora are more suggestive of a high production of 11-oxycorticosteroids.

**Dr. D. H. Johnson:**—I am very interested in the changes that Dr. Summers has demonstrated in the liver and I would like to try to link them up with the long-continued administration of nor-adrenaline. Centrilobular liver cells normally have a relatively precarious oxygen supply. Under anaerobic or partially aerobic conditions the liver produces the vaso-depressor material described by Shorr, Zweifach and Furchgott (1945) which may be responsible for the irreversible stage of shock. Further, Delorme (1951) showed that the resistance of dogs to experimental shock by sustained haemorrhagic hypotension is greatly enhanced by maintaining an adequate supply of oxygenated arterial blood to the liver.

Dr. Grayson and I (1951) have for some time past been working on the problems of blood flow in the rat's liver. We have shown that nor-adrenaline produces unequivocal decrease in liver blood flow and that this decrease also occurs when the portal supply is occluded. In other words nor-adrenaline has a marked vasoconstrictor effect on the hepatic arterioles. This vasoconstrictor effect is more marked than that produced by the equivalent pressor dose of adrenaline.

Appreciating that the nor-adrenaline infusion, continued for some forty-eight hours, was given largely as substitution therapy and that during the initial period of hypotension before nor-adrenaline was given the liver may well have sustained irreparable damage, I would submit that the value of such therapy is questionable.

**Professor F. T. Hewer:**—This adrenal tumor was evidently present from June 1945—a history of six-and-a-half years. Although these tumours notoriously invade the veins, such extension seems to have been a late event in this case.

### REFERENCES

Delorme, E. J. (1951). *Lancet*, 1, 259.
Grayson, J., and Johnson, D. H. (1952). *Brit. Med. J.* **1**, 546
Shorr, E., Zweifach, B. W. and Furchgott, R. F. (1945). *Science* **102**, 489.
Walters, Waltman. (1952). *Lancet*, 1, 221.

## Figures and Tables

**Figure f1:**